# The Mechanism Underlying the Increase in Bread Hardness in Association with Alterations in Protein and Starch Characteristics During Room-Temperature Storage

**DOI:** 10.3390/foods13233921

**Published:** 2024-12-04

**Authors:** Huaiwen Wang, Wei Liu, Peipei Zhang, Xijun Lian

**Affiliations:** 1 Tianjin Key Laboratory of Refrigeration Technology, Tianjin University of Commerce, Tianjin 300134, China; wanghw@tjcu.edu.cn (H.W.); zhangpp@tjcu.edu.cn (P.Z.); 2 Institute of Collaborative Innovation in Great Health, College of Biotechnology and Food Science, Tianjin University of Commerce, Tianjin 300134, China; 120220366@stu.tjcu.edu.cn

**Keywords:** bread hardness, staling, FTIR, ^13^C solid-state NMR, X-ray diffraction

## Abstract

Hardness constitutes one of the primary performance indices of bread. However, there is scarce literature regarding the study of the mechanisms of increased hardness in different breads. In this paper, the hardness and retrogradation rates of five popular brands of bread (DaliGarden, Mankattan, MianLunSi, TOLY, and ZhengMao) in China during storage at room temperature were determined, and the mechanism of increased hardness was revealed by the results in terms of Fourier transform infrared spectroscopy (FTIR), disulfide bonds, ^13^C solid-state nuclear magnetic resonance (NMR), X-ray diffraction, and differential scanning calorimetry (DSC). The results showed that the sequence for the degree of hardness increase among the five bread brands was DaliGarden > TOLY >Mankattan > MianLunSi > ZhengMao. The bread hardness was likely associated with the gliadin content; the more gliadin, the higher the hardness of the bread. All bread hardness values underwent a rapid increase during storage at room temperature. The hardness level of the bread preferred by Chinese individuals was approximately 105 g, and the hardness of the TOLY bread underwent relatively minor changes during storage at room temperature. The disulfide bond content of all breads apart from Mankattan decreased during storage at room temperature. The increase in the hardness of the bread was attributed to the ordered configuration of the amylopectin structures resulting from water evaporation. The results given in this paper offer a practical hardness index to control the quality of bread. This study is expected to contribute to better quality control and optimization in bread production, enhancing consumers’ satisfaction and extending products’ shelf lives.

## 1. Introduction

Bread is a massively popular and indispensable food in the human diet worldwide. It is well known that bread staling at room temperature greatly reduces its shelf life and causes 5–10% of the worldwide bread production losses every year [1]. The quality attributes, particularly the texture and hardness, are critical to consumer acceptance [2]. The increase in bread hardness during storage is a well-documented phenomenon. Understanding the mechanisms underlying these changes is essential in improving bread’s quality and extending its shelf life.

The bread hardness preferred by different nations with identical characteristics is different: 5~19 N/m^2^ (510~1938 g/m^2^) for Brazilians [3], 3~15 N/m^2^ (306~1530 g/m^2^) for Turks [4], 6~9 N/m^2^ (612~918 g/m^2^) for Spaniards [5], 400~1300 g for Portuguese [6], and 40~90 N/m^2^ (4080~9180 g/m^2^) for Australians [7]. Despite its importance, there is limited research on the hardness characteristics of popular bread brands. This gap is particularly evident in Chinese bread, which exhibits unique properties due to its distinct ingredients and processing methods. The fluctuating baking and functional nature of Chinese bread wheat is ascribed to the quality and quantity of gluten. Chinese cultivars exhibit acceptable protein content (11.1–13.4%), medium to strong dough strength, and medium to poor dough extensibility [8,9]. The amylopectin and gluten present in bread exert a significant influence on the hardness of bread. Rice with shorter amylopectin chains is a promising material for slow-hardening rice bread [10]. In a study, the hardness of bread roll crumbs increased dramatically from ~1.5 N (3 d) to ~4.0 N (7 d), which was related to gluten network changes and the staling of starch [11]. Amylopectin retrogradation is known to be a major contributor to staling [12], so alterations in its properties exert a significant influence on the hardness of bread.

However, few papers report the hardness of different brands of bread in China. The hardness increase in bread during storage is obvious, and many compounds, such as branching enzymes [13], high-amylose flour [14], and maltogenic amylase [15], have been used to hinder it. Theoretical frameworks in bread science often emphasize the roles of starch retrogradation and protein interactions as the primary factors influencing bread’s texture. Starch retrogradation contributes to the crystallization of amylopectin. However, the interplay between these components remains inadequately explored, especially in Asian bread varieties.

Research using techniques like Fourier transform infrared spectroscopy (FTIR) and nuclear magnetic resonance (NMR) has provided insights into the molecular changes during bread staling. For instance, studies have linked changes in protein secondary structures to textural variations [16]. Existing studies have typically centered on Western bread, neglecting the variations present in Chinese brands. Moreover, while some research has addressed the effects of storage on bread hardness, comprehensive analyses utilizing advanced techniques such as FTIR, disulfide bond analysis, ^13^C solid-state NMR, X-ray diffraction, and differential scanning calorimetry (DSC) are sparse. Specifically, there is a lack of comprehensive studies examining the changes in protein secondary structures and their impact on bread hardness in Chinese bread brands. The mechanisms of the increase in bread hardness during storage remain ambiguous.

In this study, the hardness and retrogradation rates of five popular brands of bread (DaliGarden, Mankattan, MianLunSi, TOLY, and ZhengMao) in China during storage at room temperature were determined, and the nature of the hardness increases in the bread was revealed via the results of FTIR, disulfide bond analysis, ^13^C solid-state NMR, X-ray diffraction, and DSC. This study is expected to contribute to better quality control and optimization in bread production, enhancing consumers’ satisfaction and extending products’ shelf lives.

## 2. Materials and Methods

### 2.1. Materials

DaliGarden toast bread slices were manufactured by the Dali Food Group Co., Ltd. (Quanzhou, China). Mankattan toast slices were manufactured by the Mankattan Industrial Co., Ltd. (Shanghai, China). ZhengMao toast slices were manufactured by the Weifang Leford Food Co., Ltd. (Weifang, China). MianLunSi toast bread slices were manufactured by the Weifang Deyeyuan Food Co., Ltd. (Weifang, China). TOLY toast bread slices were manufactured by the Shanghai TOLY Food Co., Ltd. (Shanghai, China). Other chemical reagents were obtained from the Tianjin Fuyu Fine Chemical Co., Ltd. (Tianjin, China). Tris, glycine, disodium EDTA, urea, and DTNB reagents were purchased from the Beijing Solarbio Technology Co., Ltd. (Beijing, China).

### 2.2. Treatment of Bread

Since experiments have demonstrated that low temperatures can expedite the retrogradation of starch products [17], all breads were sealed and stored at room temperature (20~26 °C). On specific dates, breads from different brands were taken out to measure the hardness, retrogradation rate, and disulfide bond content. The samples used to determine the disulfide bond content and for FTIR, ^13^C solid-state NMR, X-ray diffraction, and DSC were dried in an oven at 60 °C to a constant weight and pulverized and sieved through a 100-mesh screen.

### 2.3. Hardness Determination of Bread

The bread hardness test was performed according to the method described in reference [5]. The bread hardness of the sample was determined using a physical property analyzer (TA XT Plus, Stable Micro Systems, Godalming, UK). The P/18 probe was selected for the texture profile analysis (TPA) method. The pre-test speed, test speed, and post-test speed were 5 mm/s. The test time was set to 5 s, with a deformation strain of 40%. The trigger type was set to automatic (force trigger) in 5 g. Three replicates were performed in each case and the results were averaged.

### 2.4. Determination of Retrogradation Rate

Samples weighing 3 g (denoted as *m*_1_) were first mixed with 9 mL of deionized water and stirred to dissolve the sample fully. This step aimed to evenly distribute the sample in the aqueous phase, facilitating the subsequent enzymatic hydrolysis. Then, 0.5 mL high-temperature amylase was added to hydrolyze the non-retrograded starch in the bread at 90 °C for 2 h in a water bath. The purpose of this process was to hydrolyze the non-retrograded starch in the bread using high-temperature amylase, with the water bath providing the optimal temperature to ensure enzyme activity. After enzymatic hydrolysis, the solutions were centrifuged at 3000× *g* for 10 min to remove water-soluble hydrolysates, leaving behind precipitates, which mainly consisted of retrograded starch. The obtained precipitates were washed and centrifuged with deionized water three times to obtain the mass. Subsequently, the washed precipitates were dried in an oven at 60 °C to a constant weight, and the final dried weight was recorded (denoted as *m*_2_). Finally, the retrogradation rate was calculated according to the following formula [18]:

Retrogradation rate (%) = (*m*_2_/*m*_1_) × 100% (1)

where *m*_1_ represents the initial sample weight, and *m*_2_ represents the mass of retrograded starch after drying. This calculation determines the proportion of retrograded starch in the bread sample, allowing for an assessment of the extent of starch retrogradation.

### 2.5. Determination of Disulfide Bond Content

The determination of the disulfide bond content in bread was performed according to reference [19]. Three reagents were first configured for subsequent testing. Solution A, Tris-Gly buffer (pH = 8): 10.418 g of Tris, 6.756 g of glycine, and 1.489 g of disodium EDTA were dissolved into 1000 mL of deionized water. Solution B, Tris-Gly-8M Urea solution: 480.48 g of urea was added to solution A. Solution C, DTNB solution (4 mg/mL): 4 mg of DTNB reagent was dissolved in 1 mL of solution A.

Determination of free sulfhydryl groups (SH_1_): A solution of 15 mg of the sample was prepared by combining it with 50 μL of solution C and 5 mL of solution A. This mixture was then held at 25 °C for one hour, after which it was subjected to centrifugation (13,600× *g*) at 25 °C for 10 min. The absorbance of the resulting supernatant was then measured at 412 nm.

Determination of total sulfhydryl groups (SH_2_): A solution of 15 mg of the sample was prepared by combining it with 50 μL of solution C and 5 mL of solution B. This mixture was then held at 25 °C for one hour, after which it was subjected to centrifugation (13,600× *g*) at 25 °C for 10 min. The absorbance of the resulting supernatant was then measured at 412 nm.

Calculation formula for sulfhydryl groups:

SH (μmol/g) = 73.53 × A_412_ × *D*/*C* (2)

where A_412_ is the absorbance value of the sample after removing the reagent blank, *D* is the dilution factor, and *C* is the sample content.

The SS content was calculated as follows:

SS content = (SH_2_ − SH_1_)/2 (3)

All samples were tested in triplicate.

### 2.6. FTIR Spectroscopy

All breads were dried at 60 °C until a constant weight was reached. Then, they were pulverized using a high-speed crusher and passed through a 100-mesh sieve. Each bread was then mixed with KBr in a 1:60 ratio. A device was used to compress the mixture into a transparent sheet and the samples were mounted onto slide holders. Finally, sample spectral curves were obtained using an infrared spectrometer (Perkin-Elmer, Buckinghamshire, UK) in transmission mode.

The secondary structure of the protein in the bread was calculated according to the literature [19] based on the variation in the amide I band (1600–1700 cm^−1^). The wavenumbers for different secondary structures were as follows: 1650–1660 cm^−1^ for α-helices; 1612–1620 cm^−1^, 1680–1695 cm^−1^ for intermolecular β-sheets; 1625–1642 cm^−1^ for β-sheets; 1670–1680 cm^−1^ for β-turns; and 1642–1650^−1^ for random coils. The Peakfit 4.12 software was used to obtain the content of each secondary structure of protein in the breads.

### 2.7. ^13^C Solid-State NMR Spectroscopy

A JEOL ECZ600R 600 MHz spectrometer was used to obtain the ^13^C solid-state NMR spectra of all breads. The breads were first pulverized using a high-speed crusher and passed through a 100-mesh sieve. The dried bread powders were loaded into a 5 mm rotor at room temperature with a ^13^C frequency of 150.87 kHz, corresponding to a 90° pulse width of 2.4 μs. The rotation rate of the MAS was set to a value in the 15 kHz range.

### 2.8. X-Ray Powder Diffraction (XRD) Analysis

The breads were first pulverized using a high-speed crusher and passed through a 100-mesh sieve. The X-ray diffraction patterns of all breads were determined by a D/MAX-2500 Advance diffractometer (Rigaku, Tokyo, Japan). The diffractometer was set to 200 mA and 40 kV values. The diffraction angle (2θ) was scanned over a region ranging from 3° to 60° with a step size of 0.02° and a counting time of 0.8 s.

### 2.9. Differential Scanning Calorimetry (DSC) Analysis

A DSC404C Netzsch Instruments NA LLC (Burlington, MA, USA) was used to obtain the DSC of all breads. The breads were first pulverized using a high-speed crusher and passed through a 100-mesh sieve. The weight of bread powders was 5.9 mg and the heating temperature fields were in the range of 25 °C~300 °C, with a heating rate of 0.1 °C/min. The calibration of the DSC data was carried out using indium and an empty aluminum pan. DSC thermograms were used to determine the denaturation peak temperature (*T_p_*) and enthalpy values (Δ*H*). All samples were tested in triplicate.

### 2.10. Statistical Analysis

All data were expressed as the mean ± standard deviation based on three repetitions for each sample. Dunnett’s test was used to obtain the significant differences/extremely significant differences between the control and experimental groups (*p* < 0.05/0.01).

## 3. Results and Discussion

### 3.1. The Influence of Different Storage Dates on the Hardness of Different Brands of Bread

**Figure 1:**
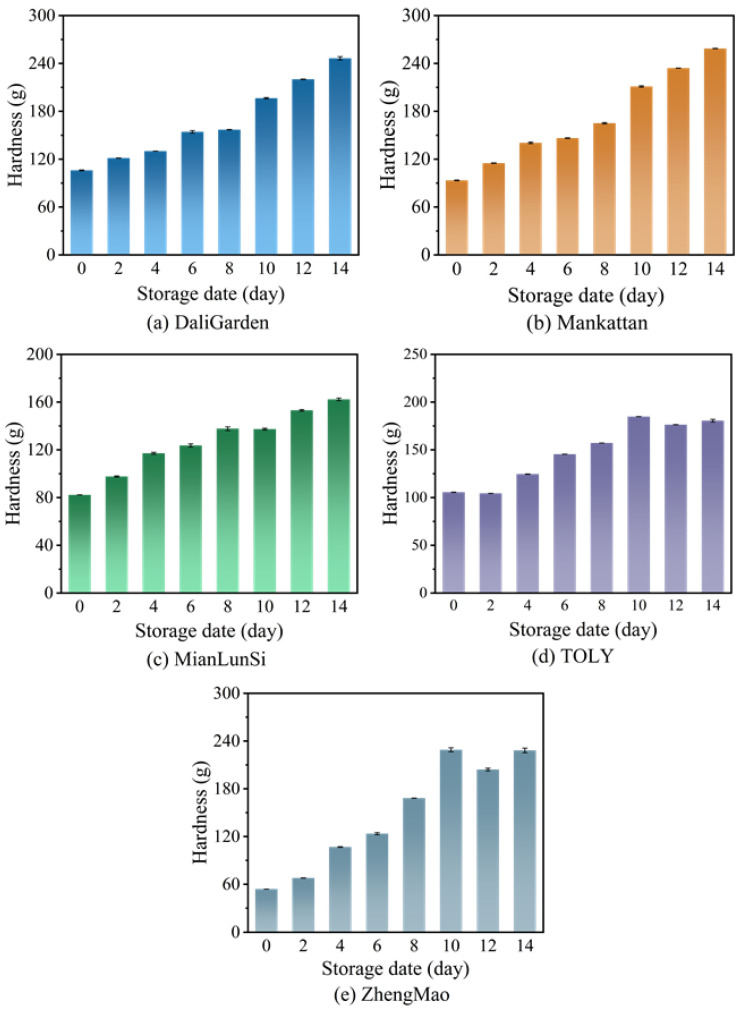
The hardness of different brands of bread at different storage dates.

Figure 1 shows the influence of different storage dates on the hardness of various brands of breads (refer to Supplementary Materials, Table S1). The results in Figure 1 indicate that the sequence regarding the hardness degrees of the five brands of bread is DaliGarden (105.97 g) > TOLY (105.62 g) >Mankattan (93.41 g) > MianLunSi (82.20 g) > ZhengMao (53.91 g). These were significantly lower than those found in Brazil [3], Turkey [4], Spain [5], Portugal [6], and Australia [7]. Chinese people tend to prefer softer bread. TOLY and DaliGarden are two of the most favored brands among Chinese consumers (https://www.sohu.com/a/730506172_120890433 (accessed on 3 August 2024)), and the hardness level of the bread preferred by Chinese people is approximately 105 g. The hardness of all breads listed in Figure 1 rises significantly as the duration of normal-temperature storage lengthens. After 14 days of storage at room temperature, the hardness of the DaliGarden, Mankattan, Mianlunsi, TOLY, and ZhengMao breads increases by 132.5%, 176.9%, 97.4%, 70.9%, and 323.3%, respectively. The hardness of the TOLY bread undergoes relatively minor changes during storage at room temperature, so it is suitable for long-term storage.

### 3.2. The Influence of Different Storage Dates on the Retrogradation Rates of Different Brands of Bread

**Figure 2:**
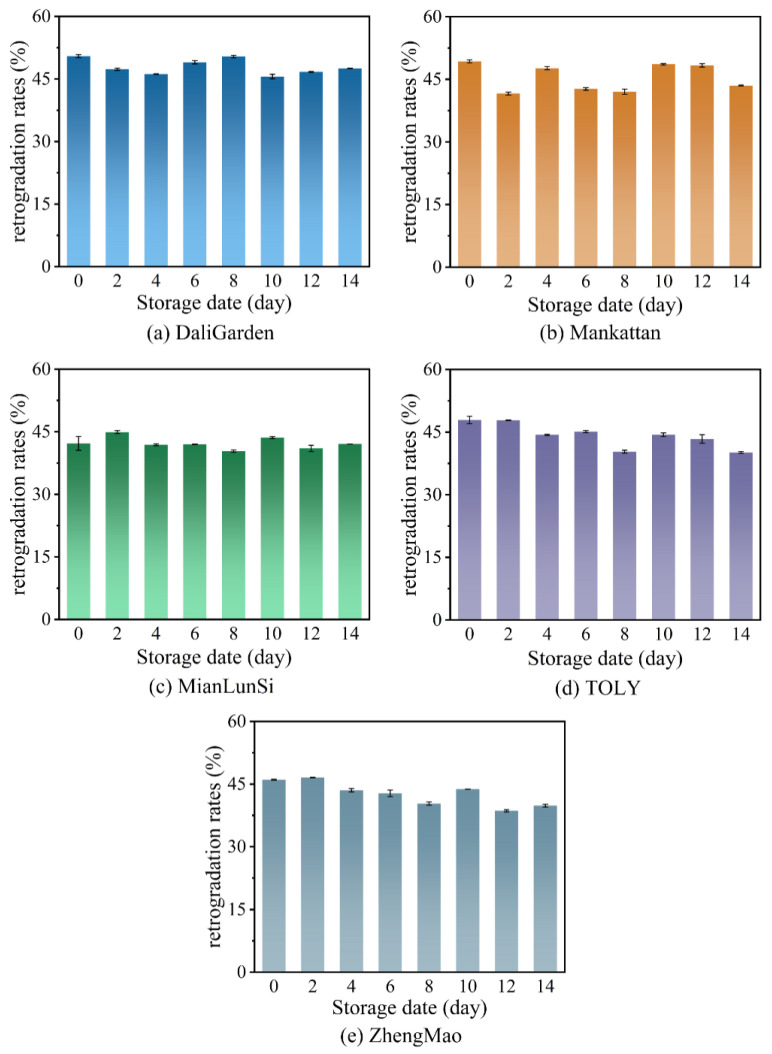
The retrogradation rates of different brands of bread at different storage dates.

Figure 2 shows the influence of different storage dates on the retrogradation rates of the different brands of bread (refer to Supplementary Materials, Table S2). The increase in bread hardness during storage is usually thought to be related to the aging of starch [17]. However, the results in Figure 2 demonstrate that the retrogradation rates of all breads remain unchanged or decrease slightly. In other words, the formation of resistant starch does not occur in all breads during storage at room temperature. This may be related to the small number of amylopectin retrogradation nuclei in the breads at room temperature. The hardness of these breads will be greater than 2000 g at cold storage. Thus, bread is not typically stored in cold storage. The increase in bread hardness at room temperature may be caused by the orderly arrangement of amylopectin, but this poorly ordered arrangement is insufficient for in vitro enzyme-resistant starch. The results in Figure 2 agree well with the reports in reference [20], suggesting that the firming of bread is controlled by multiple factors and not solely by starch retrogradation.

### 3.3. FT-IR Spectra of All Breads Stored at Room Temperature for Different Durations

**Figure 3:**
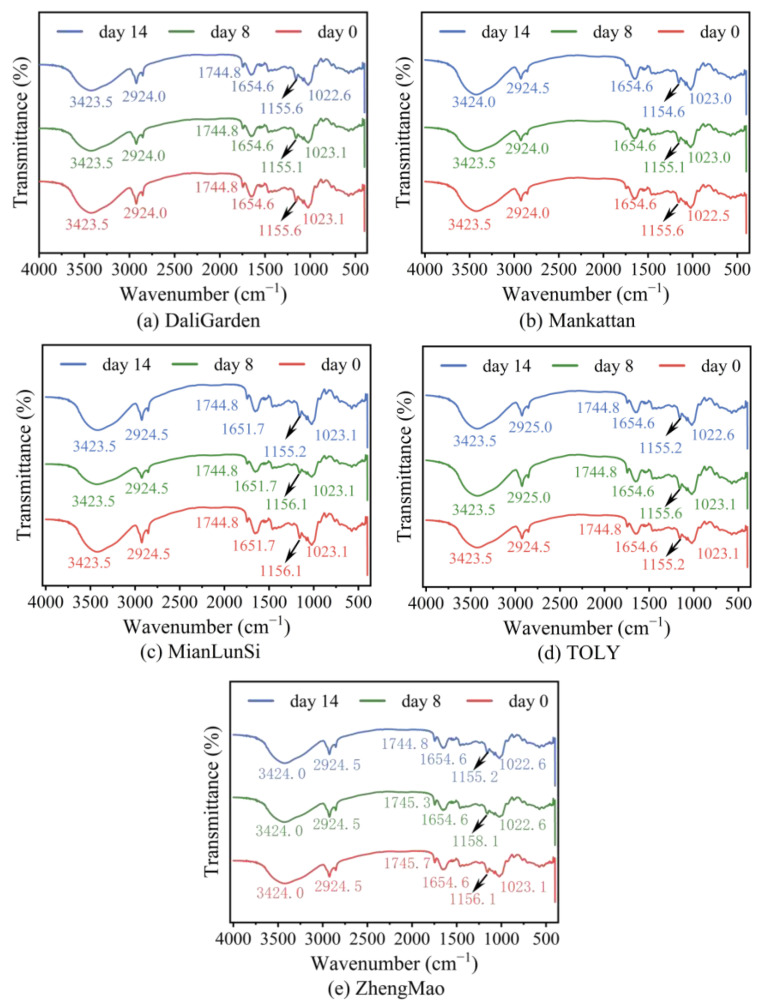
FTIR spectra of all breads stored at room temperature for different storage times.

Figure 3 shows the FTIR spectra of all breads stored at room temperature at different dates. According to references [1,21,22], the bands in Figure 3 are attributed as follows. The band around 3423 cm^−1^ corresponds to the O–H stretching vibration of wheat starch or the N–H stretching vibration of protein. The band at approximately 2924 cm^−1^ is associated with the C–H stretching vibration of methylene. The narrow band centered at 1744.8 cm^−1^ corresponds to the C=O stretching vibrations of lipids. The band at ~1654 cm^−1^ is identified as the C=O stretching vibration of the amide I band of protein. The bands at 1155 cm^−1^ are related to the C–H stretching of starch, while those at ~1023 cm^−1^ are associated with C-O-C stretching and CO (-COH) stretching vibrations, indicative of the amorphous regions of starch. The near absence of bands at 1744.8 cm^−1^ in Figure 3b indicates that the bread of Mankattan contains less oil, which can be verified by its composition. During storage, only a change in the band for oil from 1745.7 cm^−1^ to 1744.8 cm^−1^ in the ZhengMao bread is present, as seen in Figure 3e, suggesting that more wheat amyloses might be leached out in the bread and those amyloses interact with oil to form a complex (resistant starch 5). The formation of this complex contributes to the greatest increase in the hardness of this bread. For all breads, only the bands at ~1155 cm^−1^ and ~1023 cm^−1^ have a minor shift during storage, indicating that the augmentation of the bread hardness should be attributed to the alteration in the amorphous state of wheat amylopectin, rather than the formation of hydrogen bonds. However, the interaction of the gluten protein and wheat amylopectin may have an impact on this alteration.

Table 1 and Table 2 show the secondary structures of the proteins and the disulfide bond content in different brands of bread during storage. The presence of α-helices in the softer breads of ZhengMao and MianLunSi in Table 1 indicates the existence of more gliadin fractions in these breads, and the elevated gliadin levels lower the bread hardness [23,24,25]. The largest number of α-helix structures is related to the lowest level of bread hardness, and more α-gliadin might be present in the ZhengMao bread, while there are more ω-gliadins in Mianlunsi [25]. During storage, these α-helix structures are transformed into β-sheets along with the increase in bread hardness, and a correlation might be present between the β-sheet content and bread hardness. For the breads from other brands, there is no notable correlation between the content of protein secondary structures and the hardness of the bread.

The results in Table 2 show that the disulfide bond content in all bread besides Mankattan decrease significantly during storage from day 0 to day 14, especially for the TOLY bread, from 0.16 to 0.03 μmol/g. This is in accordance with the decreased content of intermolecular β-sheets (from 40.28% to 29.77%) presented in Table 3. Moreover, the increase in the structure for Mankattan from 5.81% to 86.46% in Table 3 during storage from day 8 to day 14 corresponds to the enhanced disulfide bond content from 0.22 to 0.29 μmol/g in Table 2. The intermolecular β-sheets of gluten in bread might promote the orderly arrangement of amylopectin through the adsorption of water attached to amylopectin, thus leading to the hardness increase in the bread. The inconsistency between the changes in the secondary structure of the protein in bread and the changes in bread hardness in Table 1 and Figure 1 suggests that the gluten protein in bread indirectly affects the bread hardness through changes in the starch structure.

### 3.4. The ^13^C Solid-State NMR Spectra of All Breads Stored at Room Temperature for Different Times

**Figure 4:**
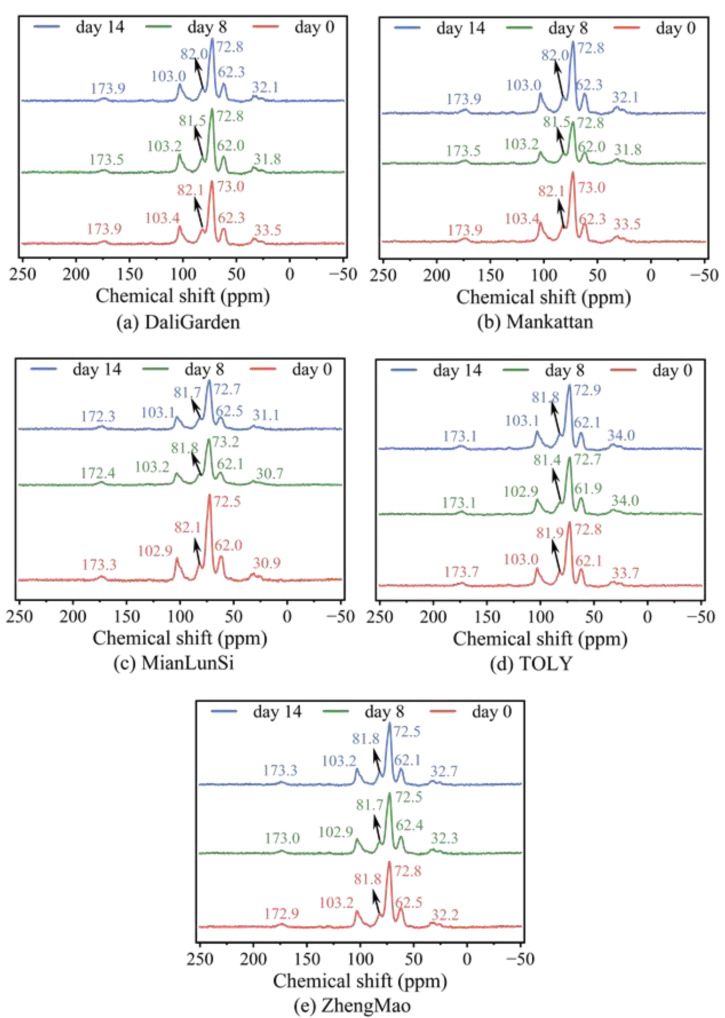
^13^C solid-state NMR spectra of all breads stored at room temperature for different storage times.

Figure 4 shows the ^13^C solid-state NMR spectra of all breads stored at room temperature at different dates. According to references [26,27,28,29,30], the resonances at ~103, ~82, ~73, and ~62 ppm in Figure 4 correspond to C1, C4, C2/C3/C5, and C6 of wheat starch in bread, respectively. There are only two resonances assigned to protein in bread in Figure 4: ~173 ppm for the backbone CO of polypeptides and 35~30 ppm for the Gln *γ* (Q*_γ_*)/Pro *β* (P*_β_*) of gluten in bread. All resonances undergo slight alterations, with irregularity during storage at room temperature. After meticulous analysis, it was discovered that, for the ZhengMao and Mankattan breads, with a greater hardness increase during storage, the resonance changes in C2, C3, and C5 of wheat starch in the bread exhibit a positive correlation with the hardness alteration. Their resonances at this point decrease from 72.8/73.0 ppm to 72.5/72.8 ppm, respectively, during storage. Since the hydrogen bonds of the bread remain relatively unchanged during storage, as seen in Figure 3, it is hypothesized that the increase in bread hardness might be associated with alterations in the three-dimensional structure of the hydrogen bonds at the C2, C3, and C5 carbon atoms of wheat amylopectin.

### 3.5. X-Ray Diffraction of All Breads Stored at Room Temperature for Different Times

**Figure 5:**
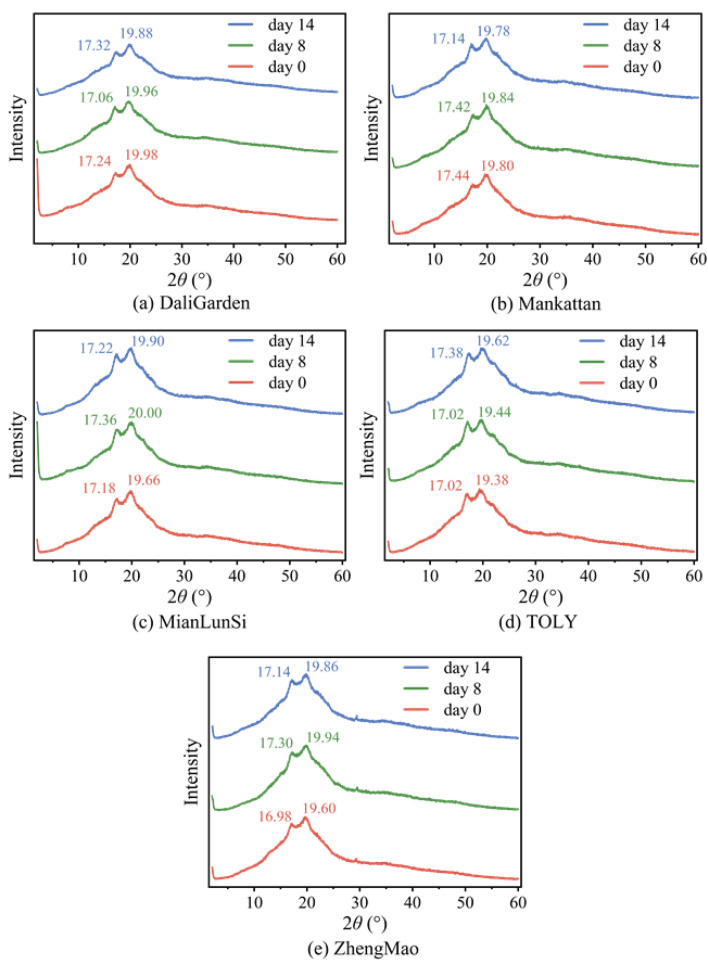
X-ray diffraction patterns of all breads stored at room temperature for different storage times.

The XRD patterns of all breads stored at room temperature for different durations are shown in Figure 5. The diffraction angles (2θ) of all breads were at 2θ ~17° and ~20°, which are different from those of wheat starch, with the diffraction angles (2θ) of 15°, 17°, 18°, and 23° [30,31]; amylose–lipid complexes (2θ at 13.2° and 19.8°) [32]; retrograded wheat starch (2θ at 7.3°, 13.0° and 19.8°) [33]; or wheat amylose (2θ at 17.1° and 22.4°) [34]. They are nearly the same as those of wheat amylopectin, which has diffraction angles (2θ) of 13.2°, 17.1°, and 20.0° [34]. The absence of diffraction angles for wheat starch suggests that the original crystal structure of the wheat granules is destroyed in the bread-making process, although intact starch granules are still present in the bread [35]. During storage at room temperature, for all breads, their X-ray diffraction peak intensities at the diffraction angle of ~20° are enhanced. Such results suggest that the main reason for the hardness increase in bread during storage lies in the enhanced ordered arrangement of macromolecules of wheat amylopectin based on the formation of hydrogen bonds at C2, C3, and C5. The most prominent finding in Figure 5 is that, compared with the DaliGarden bread in Figure 5a, the intensity of the X-ray diffraction angle at ~17° for the TOLY bread is higher than that of the DaliGarden bread, and the TOLY bread exhibits a smaller increase in hardness during storage. Therefore, this peak can be seen as a sign of a slow increase in bread hardness. Then, a speculation about the bread hardness alteration during storage can be made. There are two types of wheat amylopectin leached out from granules during bread preparation, like maize amylopectin before and after co-crystallization with NaCl in reference [18], as well as snowflake- and rod-shaped ones. Snowflake and radial wheat amylopectin form crystals corresponding to two peaks with diffraction angles of ~17° and ~20°, respectively. The former, with a loose arrangement, causes higher bread hardness, and the latter, with a tight arrangement, leads to lower values. More *α*-gliadins promote the arrangement of snowflake wheat amylopectin and lead to soft bread [25], and more *ω*-gliadins enhance the arrangement of rod-like wheat amylopectin to produce hard bread [25,36]. Therefore, the control of bread hardness could be achieved by screening wheat flour with a certain amount of α-gliadins/*ω*-gliadins or the addition of *α*-gliadin/*ω*-gliadin fractions. Table 3 shows the influence of different storage dates on the thermal properties of other brands of bread. The results in Table 3 indicate that the peak denaturation temperature (*T*p) and enthalpy (Δ*H*) of all breads remained almost unchanged during storage at room temperature for 14 days, verifying that the bread hardness increase originates from the rearrangement of wheat amylopectin but not the formation of new crystals of retrograded starch.

## 4. Conclusions

The mechanisms behind the hardness increase in five different brands of bread in China during storage at room temperature were experimentally investigated. The hardness level of the bread preferred by Chinese individuals is approximately 105 g, which is considerably lower than that preferred by those from countries outside China. The disulfide bond content of all breads apart from Mankattan decreased during storage at room temperature. The intensity of the X-ray diffraction angle (2θ) at ~17° can be seen as a sign of a slow increase in bread hardness. The increase in bread hardness during storage is attributed to the rearrangement of wheat amylopectin molecules, and the structure of wheat amylopectin, as well as the content of gluten protein, exert a significant influence on the increase in bread hardness. These research results are expected to aid in improving quality control and optimization in bread production, thereby increasing consumers’ satisfaction and prolonging the shelf lives of products.

## Figures and Tables

**Table 1:** The influence of different storage dates on the secondary structure content of different brands of bread (%).

Sample	α-Helix	Intermolecular *β*-Sheet	Intra-Molecular Aggregation Extended *β*-Sheet	*β*-Turn	Random Coil
DaliGarden day 0	0	17.36	17.20	7.58	57.86
DaliGarden day 8	0	41.05	46.53	12.41	0
DaliGarden day 14	0	15.14	16.83	8.47	59.55
Mankattan day 0	0	0	22.00	78.00	0
Mankattan day 8	0	5.81	79.14	15.05	0
Mankattan day 14	0	86.46	13.54	0	0
MianLunSi day 0	81.05	0	18.95	0	0
MianLunSi day 8	0	4.16	84.68	11.16	0
MianLunSi day 14	0	43.02	47.53	9.45	0
TOLY day 0	0	40.28	59.72	0	0
TOLY day 8	0	35.16	64.84	0	0
TOLY day 14	0	29.77	70.23	0	0
ZhengMao day 0	83.73	0	16.27	0	0
ZhengMao day 8	0	0	12.79	87.21	0
ZhengMao day 14	0	35.62	64.38	0	0

**Table 2:** The influence of different storage dates on the disulfide bond content of different brands of bread (μmol/g).

Sample	Storage Date (Day)		
	0	8	14
DaliGarden	0.61 ± 0.01	0.53 ± 0.00 *	0.36 ± 0.01 **
Mankattan	0.31 ± 0.02	0.22 ± 0.00 *	0.29 ± 0.01 *
MianLunSi	0.16 ± 0.01	0.07 ± 0.01 *	0.03 ± 0.02
TOLY	0.53 ± 0.03	0.12 ± 0.01 **	0.05 ± 0.03
ZhengMao	0.45 ± 0.02	0.15 ± 0.03 *	0.13 ± 0.03

Note: Values followed by * indicate significance levels, with * denoting *p* < 0.05 and ** denoting *p* < 0.01. Data are presented as means ± SD (*n* = 3).

**Table 3:** The influence of different storage dates on the thermal properties of different brands of bread.

Sample	*T*p_1_ (°C)	Δ*H*_1_ (J/g)	*T*p_2_ (°C)	Δ*H*_2_ (J/g)
DaliGarden day 0	104.40 ± 0.09	77.69 ± 1.82	216.84 ± 0.09	13.34 ± 1.17
DaliGarden day 8	107.15 ± 0.17 **	73.17 ± 6.48	215.93 ± 0.34	13.26 ± 0.67
DaliGarden day 14	101.30 ± 0.52 **	83.63 ± 0.40	216.27 ± 6.47	18.8 ± 2.12
Mankattan day 0	101.61 ± 0.33	96.43 ± 2.39	210.49 ± 0.43	15.98 ± 0.60
Mankattan day 8	100.97 ± 1.67	86.41 ± 0.22	208.08 ± 0.00	20.71 ± 0.70 *
Mankattan day 14	102.45 ± 0.15	77.10 ± 0.08 **	208.29 ± 0.30	23.23 ± 0.61
MianLunSi day 0	99.97 ± 0.01	63.25 ± 2.08	209.72 ± 1.00	24.14 ± 0.10
MianLunSi day 8	100.32 ± 1.00	56.89 ± 1.05	207.41 ± 0.16	27.82 ± 0.11 **
MianLunSi day 14	97.95 ± 1.32	79.19 ± 9.15	208.29 ± 0.10 *	28.56 ± 0.79
TOLY day 0	106.38 ± 0.59	63.04 ± 0.68	210.46 ± 0.44	28.47 ± 0.13
TOLY day 8	106.12 ± 0.20	60.62 ± 0.04	211.69 ± 0.39	27.68 ± 0.70
TOLY day 14	104.24 ± 1.58	75.6 ± 2.40 *	211.94 ± 0.17	29.6 ± 1.61
ZhengMao day 0	100.31 ± 0.33	58.57 ± 0.88	212.49 ± 0.27	24.18 ± 1.57
ZhengMao day 8	99.47 ± 1.16	73.88 ± 9.81	213.48 ± 0.08	19.27 ± 0.15
ZhengMao day 14	97.63 ± 1.01	70.22 ± 0.37	214.39 ± 0.20	19.61 ± 3.07

Note: Values followed by * indicate significance levels, with * denoting *p* < 0.05 and ** denoting *p* < 0.01. Data are presented as means ± SD (*n* = 3).

## Data Availability

The original contributions presented in the study are included in the article/Supplementary Materials, further inquiries can be directed to the corresponding author.

## Supplementary Materials

The following supporting information can be downloaded at: https://www.mdpi.com/article/10.3390/foods13233921/s1, Table S1: The influence of different storage date on the hardness of different brands of breads (g); Table S2: The influence of different storage date on the retrogradation rates of different brands of bread (%).
