# Supplementary material for: The Mechanism Underlying the Increase in Bread Hardness in Association with Alterations in Protein and Starch Characteristics During Room-Temperature Storage
Source: Foods. 2024 Dec 4;13(23):3921. doi: 10.3390/foods13233921 (PMC11641183; doi:10.3390/foods13233921)
Supplement: Supplementary file 1 [file foods-13-03921-s001.zip › foods-3324321-supplementary.pdf]

Supplementary material

Table S1. The influence of different storage date on the hardness of different brands of breads (g).

Samples	Storage date (day)							
	0	2	4	6	8	10	12	14
DaliGarden	105.97±0.51	121.33±0.19**	130.07±0.05**	154.21±1.57**	156.90±0.30	196.39±0.82**	220.04±0.37**	246.41±2.013**
Mankattan	93.41±0.47	115.01±0.38**	140.49±0.97**	146.34±0.47*	165.00±0.85**	211.12±0.93**	234.10±0.16**	258.63±0.28**
MianLunSi	82.20±0.10	97.68±0.48**	117.10±0.81**	123.65±1.33	137.71±1.48*	137.33±0.74	153.09±0.68**	162.274±1.07*
TOLY	105.62±0.12	104.45±0.08*	124.58±0.16**	145.38±0.10**	157.15±0.05**	184.79±0.09**	176.32±0.19**	180.53±1.39
ZhengMao	53.91±0.03	67.82±0.21**	106.85±0.41**	123.63±1.33**	168.21±0.17**	228.98±2.50**	204.13±1.68*	228.22±2.88*

Note: Values followed by * indicate significance levels, with * denoting p<0.05 and ** denoting p<0.01. Data are presented as means ± SD (n=3).

Table S2. The influence of different storage date on the retrogradation rates of different brands of bread (%).

Samples	Storage date (day)							
	0	2	4	6	8	10	12	14
DaliGarden	50.46±0.36	47.30±0.28*	46.17±0.08*	49.00±0.40*	50.36±0.29	45.57±0.54*	46.70±0.09	47.54±0.04*
Mankattan	49.29±0.37	41.60±0.35**	47.66±0.38**	42.70±0.32**	42.02±0.60	48.63±0.16**	48.34±0.39	43.51±0.13**
MianLunSi	42.21±1.66	44.91±0.35	41.88±0.23*	42.02±0.07	40.34±0.28*	43.57±0.25*	41.01±0.77	42.06±0.03
TOLY	47.90±0.88	47.86±0.08	44.35±0.15**	45.12±0.27	40.30±0.35**	44.37±0.43*	43.37±1.01	40.14±0.21
ZhengMao	46.04±0.13	46.58±0.06	43.53±0.39*	42.78±0.77	40.34±0.39	43.78±0.04*	38.59±0.26**	39.83±0.33*

Note: Values followed by * indicate significance levels, with * denoting p<0.05 and ** denoting p<0.01. Data are presented as means ± SD (n=3).
